# Late Gadolinium Enhancement Magnetic Resonance Imaging (MRI) for Predicting Left Ventricular Reverse Remodeling in Non-Ischemic Cardiomyopathy: A Systematic Review and Meta-Analysis

**DOI:** 10.3390/jcm14030895

**Published:** 2025-01-29

**Authors:** Yuri Teraoka, Shingo Kato, Naofumi Yasuda, Shungo Sawamura, Nobuyuki Horita, Daisuke Utsunomiya

**Affiliations:** 1 Department of Diagnostic Radiology, Graduate School of Medicine, Yokohama City University, Kanagawa 236-00204, Japanyasuda.nao.mz@yokohama-cu.ac.jp (N.Y.); sawa0808@yokohama-cu.ac.jp (S.S.);; 2 Chemotherapy Center, Yokohama City University Hospital, Kanagawa 236-0004, Japan

**Keywords:** non-ischemic cardiomyopathy, cardiac MRI, late gadolinium enhanced MRI, left ventricular reverse remodeling, meta-analysis

## Abstract

**Background/Objectives**: Late gadolinium enhancement (LGE)-MRI has proven utility in prognosticating outcomes in patients with non-ischemic cardiomyopathy (NICM). However, evidence regarding its ability to predict responsiveness to optimal medical therapy remains insufficient. This study conducted a meta-analysis to evaluate the predictive utility of LGE-MRI for left ventricular reverse remodeling (LVRR) in response to pharmacological therapy. **Methods**: Data from 1092 NICM patients across 13 studies were included in the analysis. To assess the predictive ability of LGE-MRI for LVRR following optimal medical therapy, a pooled odds ratio was calculated using an inverse variance random-effects meta-analysis. Subgroup analyses were performed by stratifying patients based on the presence or absence of left ventricular dilation and by LVEF (<30% vs. ≥30%). **Results**: The pooled odds ratio of the absence of LGE for predicting LVRR in NICM was 3.72 (95% CI: 2.83–4.90, I^2^ = 0, P for heterogeneity = 0.54). A comparison of pooled odds ratios between dilated cardiomyopathy (DCM) and NICM showed no significant difference (*p* = 0.16). A subgroup analysis in NICM based on the left ventricular ejection fraction (LVEF) demonstrated no significant difference in odds ratios between patients with LVEF <30% (OR: 2.96, 95% CI: 1.80–4.87) and those with LVEF ≥30% (OR: 3.97, 95% CI: 2.97–5.31), (*p* = 0.13). **Conclusions**: This meta-analysis suggested that LGE-MRI serves as a reliable predictor of LVRR in patients with NICM, regardless of left ventricular dilation or baseline LVEF classification.

## 1. Introduction

Late gadolinium enhancement (LGE) in cardiac magnetic resonance imaging (MRI) has emerged as a pivotal tool in the assessment and management of patients with non-ischemic cardiomyopathy (NICM) [1,2]. LGE is strongly associated with an increased risk of all-cause mortality, heart failure hospitalization, and sudden cardiac death [3,4,5]. Previous studies have demonstrated that the presence of LGE is significantly correlated with higher annual event rates compared to LGE-negative patients [6]. These findings highlighted the importance of LGE as a prognostic marker that enables the identification of high-risk patients and facilitates the development of individualized management strategies.

Beyond its role as a prognostic marker, LGE serves as a valuable tool for therapeutic decision-making in NICM [7,8,9]. Patients with multiple or extensive LGE lesions are more likely to exhibit poor left ventricular reverse remodeling (LVRR) in response to optimal medical therapy, making LGE a critical factor in guiding treatment decisions [10]. However, compared to its prognostic value, evidence regarding its predictive utility for LVRR remains limited and inconsistent, with some studies reporting conflicting data. Therefore, it is imperative to validate the utility of LGE in this context through a comprehensive meta-analysis. This study aims to systematically evaluate the role of LGE in predicting responsiveness to medical therapy in NICM patients, providing a robust evidence base for its clinical application.

## 2. Materials and Methods

### 2.1. Search Strategy and Selection Criteria

We adopted the methodology proposed by the Cochrane Collaboration and adhered to the 2020 guidelines for systematic review and meta-analysis reporting (PRISMA) [11]. A comprehensive database search was conducted on 20 November 2024, using PubMed, Web of Science, and the Cochrane Library. The following keywords were used: “non-ischemic dilated cardiomyopathy”, “DCM”, “left ventricular reverse remodeling”, and “LVRR”, among others (Supplementary Material File S1). The inclusion criteria encompassed all articles that could be identified using the specified keywords and reported on the presence of LGE-MRI or quantitative fibrosis markers as predictors of the incidence of LVRR following optimal medical therapy for non-ischemic cardiomyopathy. The exclusion criteria included case reports, animal studies, and non-English articles. Two reviewers (Y.T. and S.K.) screened all titles and abstracts from the search results and conducted a full review of studies that were potentially relevant. Discrepancies were resolved by a third reviewer. As this study was a meta-analysis and did not involve clinical patient information, institutional review board approval was deemed unnecessary. This study protocol was not registered in any online database.

### 2.2. Outcome Assessment

The primary outcome of this meta-analysis was to evaluate the predictive ability of LGE-MRI presence or quantitative fibrosis markers for the occurrence of LVRR after optimal medical therapy in non-ischemic cardiomyopathy. Two reviewers (Y.T. and S.K.) extracted the following study characteristics: author names, publication year, country of origin, patient disease, age, gender, MRI parameters (the presence of LGE and indicators for quantitative evaluation of LGE), and LVRR frequency.

### 2.3. Assessment of Risk of Bias

The risk of bias was assessed using the Newcastle-Ottawa Quality Assessment Scale and case-control studies [12]. To evaluate publication bias, funnel plots were generated for the predictive ability (odds ratio) of the presence of LGE-MRI or quantitative fibrosis markers.

### 2.4. Data Integration and Statistical Analysis

A meta-analysis was conducted using RevMan 5.41 (Cochrane Collaboration, London, UK). Pooled odds ratios for LVRR prediction by LGE-MRI were estimated using an inverse variance method with a random-effects model. Heterogeneity across studies was evaluated using the I^2^ statistic, where 0% indicates no heterogeneity and 100% indicates substantial heterogeneity [13]. Publication bias was assessed using Begg’s test, and Kendall’s tau was calculated. A *p*-value < 0.10 for Begg’s test was considered statistically significant, while for other statistical methods, a *p*-value < 0.05 was considered statistically significant.

## 3. Results

### 3.1. Characteristics of Included Studies

**Figure 1:**
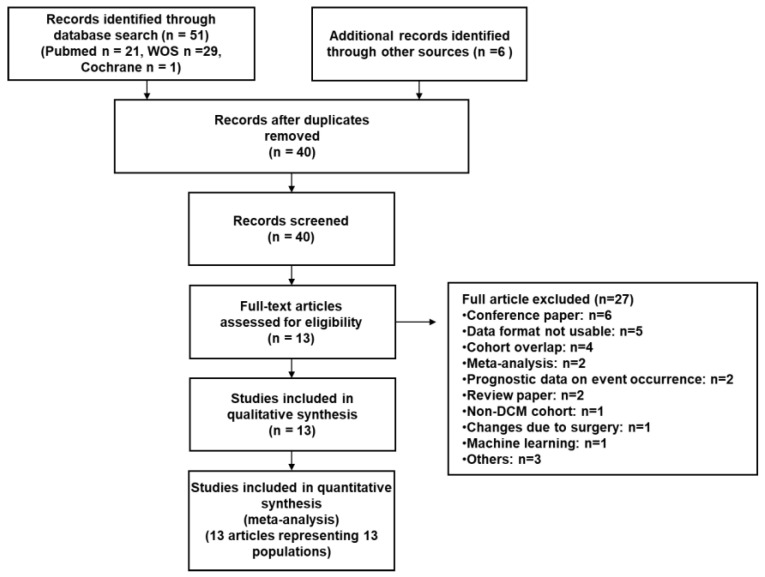
PRISMA flow diagram.

Finally, 13 eligible studies were selected from 51 candidate studies (Figure 1). Data from 1092 NICM patients across 13 studies were included in the analysis. The characteristics of these studies are summarized in Table 1. The publication years ranged from 2010 to 2023, encompassing various countries [7,14,15,16,17,18,19,20,21,22,23,24,25]. Among the selected studies, seven originated from Japan [15,16,18,19,21,22,24], two from China [23,25] and Italy [17,20], and one each from the Czech Republic [7] and South Korea [14]. Four studies included more than one-hundred patients [15,22,23,24]. Of the studies analyzed, 11 focused on dilated cardiomyopathy (DCM) with left ventricular dilation [7,15,16,17,18,19,21,22,23,24,25], while two included both cases with and without left ventricular dilation [14,20]. Furthermore, five studies reported an average left ventricular ejection fraction (LVEF) of less than 30% [7,14,19,23,25], while eight studies reported an average LVEF of 30% or higher [15,16,17,18,20,21,22,24] (Table 2).

### 3.2. Risk of Bias Assessment

The quality assessment of the studies for risk of bias is summarized in Supplementary Material File S2. Overall, 84% (11/13) of the studies were rated as high quality, achieving a score of 80% or higher on the quality scale. Meanwhile, 15% (2/13) were rated as moderate quality, scoring between 50% and 80%. None of the studies were rated as low quality, with scores below 50%. Additionally, no significant publication bias was detected in the funnel plot (Kendall’s tau = 0.1341, *p* = 0.5093) (Supplementary Material File S3).

### 3.3. Predictive Value of Late Gadolinium Enhancemen-Magnetic Resonance Imaging (LGE-MRI) for Left Ventricular Reverse Remodeling (LVRR) by Optimal Medical Therapy

**Figure 2:**
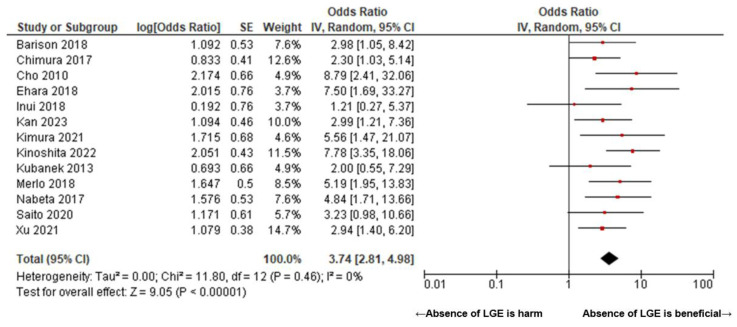
Forrest plot of pooled odds ratio of the absence of LGE for LVRR in NICM with pharmacological therapy. The pooled odds ratio was 3.74 (95% CI: 2.81–4.98, I^2^ = 0, P for heterogeneity = 0.46) [7,14,15,16,17,18,19,20,21,22,23,24,25].

**Figure 3:**
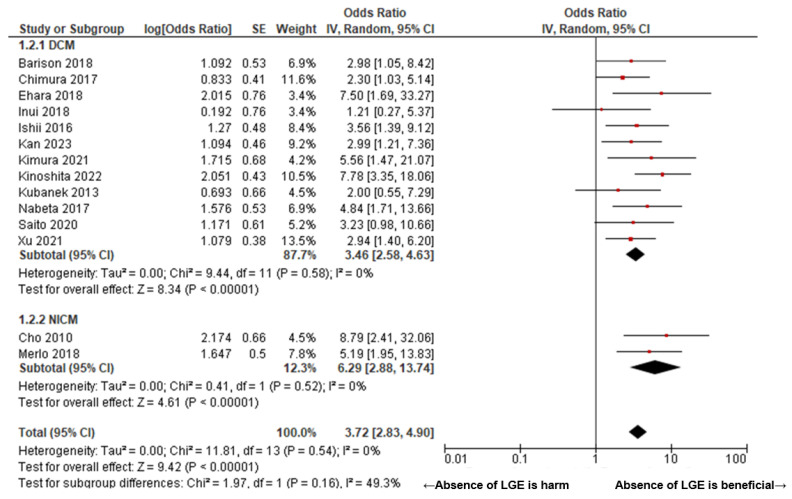
Forrest plot of the pooled odds ratio of the absence of LGE for LVRR in DCM and NICM. In DCM, the pooled odds ratio was 3.46 (95% CI: 2.58–4.63, I^2^ = 0, P for heterogeneity = 0.58), with no significant difference observed compared to the NICM cohort (*p* = 0.16). [7,14,15,16,17,18,19,20,21,22,23,24,25].

**Figure 4:**
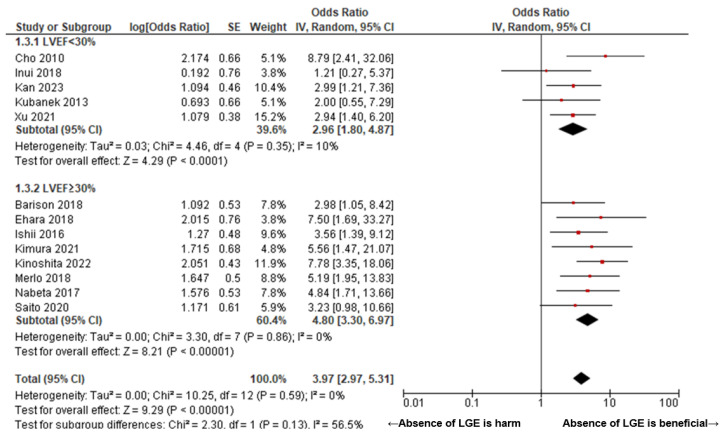
Forrest plot of the pooled odds ratio of the absence of LGE for LVRR in NICM: A subgroup analysis based on the left ventricular ejection function. In patients with LVEF <30%, the pooled odds ratio was 2.96 (95% CI: 1.80–4.87, I^2^ = 10, P for heterogeneity = 0.35), while in patients with LVEF ≥30%, the pooled odds ratio was 4.80 (95% CI: 3.30–6.97, I^2^ = 0, P for heterogeneity = 0.86). No significant difference was observed in the odds ratios between the two groups (*p* = 0.13). [7,14,15,16,17,18,19,20,21,22,23,24,25].

Figure 2 presents a Forrest plot illustrating the pooled odds ratio for predicting left ventricular reverse remodeling (LVRR) following pharmacological therapy using delayed enhancement MRI in patients with non-ischemic cardiomyopathy (NICM). The pooled odds ratio for the absence of LGE was 3.74 (95% confidence interval (CI): 2.81–4.98, I^2^ = 0, P for heterogeneity = 0.46) (Figure 2). Figure 3 compares the pooled odds ratios between two cohorts stratified based on the presence or absence of left ventricular dilation. In patients with DCM, the pooled odds ratio was 3.45 (95% CI: 2.54–4.69, I^2^ = 0, P for heterogeneity = 0.49), with no statistically significant difference observed in comparison to the NICM cohort (*p* = 0.16). A subgroup analysis based on the left ventricular systolic function in NICM demonstrated that the pooled odds ratio was 2.96 (95% CI: 1.80–4.87, I^2^ = 10, P for heterogeneity = 0.35) in patients with LVEF <30% and 4.80 (95% CI: 3.30–6.97, I^2^ = 0, P for heterogeneity = 0.86) in patients with LVEF ≥30%. No statistically significant difference was observed between these two subgroups (*p* = 0.13) (Figure 4).

## 4. Discussion

This meta-analysis demonstrates that LGE on cardiac MRI serves as a powerful predictor of LVRR in patients with NICM. The pooled odds ratio of 3.72 for the absence of LGE indicates its significant predictive value, underscoring its clinical utility. Furthermore, the lack of heterogeneity supports the reliability of LGE as a predictive factor. A comparison between DCM and NICM revealed no significant difference in predictive value. These findings affirm the consistency of LGE as a predictor of LVRR across various NICM subtypes, highlighting its role as a universal marker in diverse NICM populations.

LGE-MRI has been established as a critical prognostic tool across various cardiac diseases, reflecting the expansion of the extracellular space due to myocardial fibrosis [26,27,28,29,30]. Pathologically, LGE primarily represents focal fibrosis, which is distinct from diffuse interstitial fibrosis, such as collagen deposition in hypertensive changes, or infiltrative fibrosis due to amyloid deposition in cardiac amyloidosis [31,32]. Focal fibrosis detected by LGE has been associated with the formation of reentrant circuits leading to ventricular tachycardia, and its progression correlates with advanced heart failure. In DCM patients, LGE plays a pivotal role in guiding therapeutic decisions, including the use of implantable cardioverter defibrillators, cardiac resynchronization therapy, and ventricular tachycardia ablation [2]. With advancements in pharmacological therapy for heart failure, there is growing interest in predicting softer endpoints, such as LVRR. While prior studies have reported favorable outcomes in predicting LVRR based on LGE in patients receiving optimal medical therapy, conflicting results have also been documented. To reconcile these discrepancies, we conducted a meta-analysis to synthesize current evidence.

In our study, a subgroup analysis based on baseline LVEF confirmed that the predictive value of LGE is independent of LVEF. Patients with LVEF <30% (OR 2.96) and those with LVEF ≥30% (OR 3.97) exhibited comparable odds of LVRR in the absence of LGE (*p =* 0.13). This finding underscores the utility of LGE as a predictive tool irrespective of baseline systolic function. Collectively, these results validate LGE-MRI as a reliable and comprehensive modality for assessing myocardial viability and remodeling potential, making it an indispensable element in the stratification and management of NICM patients, regardless of left ventricular dilation or LVEF category. The insights from this study provide valuable guidance for the development of individualized therapeutic strategies aimed at optimizing outcomes in NICM patients.

Recently, non-dilated left ventricular cardiomyopathy (NDLVC) has come to be distinguished from DCM. Recent studies have comprehensively characterized these diseases using cardiac magnetic resonance imaging and genetic testing. Pathogenic variants in arrhythmia-associated genes were identified in 40% of patients with NDLVC, compared to 23% of those with DCM (*p* < 0.001). Additionally, the left ventricular ejection fraction (LVEF) was significantly higher in NDLVC (51% ± 12%) than in DCM (36% ± 15%; *p* < 0.001). Moreover, the LGE of the free wall was observed in 27% of NDLVC cases, compared to 14% in DCM (*p* < 0.001). Patients with septal LGE exhibited a significantly higher risk of sudden cardiac death or arrhythmic events (HR: 1.929; 95% CI: 1.033–3.601; *p* = 0.039), and LGE was identified as an independent prognostic factor alongside left ventricular dilation and advanced NYHA classification [33]. These findings suggest that LGE may play a pivotal role in the diagnosis and risk stratification of NDLVC, warranting further investigation into its clinical applications.

The diagnosis of NICM can sometimes be challenging, necessitating careful differential diagnosis. For instance, it is known that right ventricular remodeling in the athlete’s heart may resemble the early symptoms of ARVC [34]. Myocarditis is also a significant disease that must be differentiated from NICM. According to a large-scale study by the European Association of Cardiovascular Imaging, CMR is commonly used in the diagnosis of acute myocarditis, with non-ischemic late gadolinium enhancement frequently employed as a diagnostic criterion [35]. However, the distribution of LGE alone may make it difficult to differentiate from NICM, thus careful interpretation is required. Lastly, myocardial injuries caused by COVID-19 can also present with pathologies similar to NICM, making it an important differential diagnosis [36]. In addition, the accurate interpretation of electrocardiograms is crucial for the differential diagnosis of cardiomyopathies and for determining treatment strategies. Ventricular tachycardia, often seen in cardiomyopathies, can be mistaken for supraventricular tachycardia [37]. Therefore, accurately differentiating between these two conditions is essential for determining the appropriate indications for implantable cardioverter-defibrillator placement.

## 5. Limitations

First, as many of the studies included in this analysis are small-scale and retrospective, further validation in large-scale prospective studies is required. Second, since LGE requires the administration of gadolinium contrast agents, it cannot be evaluated in patients with renal dysfunction. In this meta-analysis, the utility of native T1 mapping, which can be performed without contrast agents, could not be evaluated [38]. Given that severe NICM often coexists with renal impairment, echocardiography and myocardial scintigraphy may play a critical role in predicting LVRR in such patient populations. Furthermore, despite remarkable advancements in pharmacological therapy for heart failure, a stratified analysis based on the type of administered medications could not be performed due to insufficient data in this study. Finally, analyzing mixed patient groups with different baseline LVEFs could have increased heterogeneity in the results. Therefore, we performed a subgroup analysis using 30% LVEF as the threshold. In both groups, those with LVEF <30% and LVEF ≥30%, the absence of LGE was a predictor of LVRR. In NICM, LGE does not correlate as closely with prognosis as it does in ischemic cardiomyopathy [39]. Prognosis is determined by a greater number of clinical factors; therefore, caution is necessary when interpreting these results.

## 6. Conclusions

This meta-analysis suggested that LGE-MRI serves as a reliable predictor of LVRR in patients with NICM, regardless of left ventricular dilation or baseline LVEF classification.

## Figures and Tables

**Table 1:** Characteristics of included studies.

Author Year	Country	Diagnosis	Diagnostic Criteria	No. of Patients	Age	Sex (Male)	Medications
Kan 2023 [25]	China	DCM	N/A	94	LVRR− n = 53: 49.30 (14.58) LVRR+ n = 41: 45.95 (14.39)	58/94	Angiotensin receptor neprilysin inhibitor, ACEI/ARB, Beta-blocker, MRA, diuretics, Sodium-glucose co-transporter inhibitors
Kinoshita 2022 [24]	Japan	NIDCM	American Society of Echocardiography	113	63 (12)	91/113	Beta-blocker, ACEI/ARB, MRA, Oral inotropic agents
Xu 2021 [23]	China	IDCM	WHO criteria	157	46 (15)	106/157	ACEI/ARB, Beta-blocker, MRA, diuretics, digoxin, warfarin
Kimura 2021 [22]	Japan	DCM	Endomyocardial biopsy	131	50.1 (11.9)	96/131	ACEI/ARB, Beta-blocker, MRA
Saito 2020 [21]	Japan	DCM	N/A	55	55.5 (13.1)	44/55	ACEI/ARB, Beta-blocker, diuretics, Aldosterone receptor antagonists
Inui 2018 [19]	Japan	DCM	-MOGE(S) -European Society of Cardiology Working Group	33	59.6 (10.7)	22/33	ACEI/ARB, Beta-blocker, diuretics
Ehara 2018 [18]	Japan	NIDCM	N/A	39	56 (14)	52/65	ACEI/ARB, Beta-blocker, MRA, diuretics
Barison 2018 [17]	Italy	NIDCM	WHO criteria	71	57 (14)	43/71	ACEI/ARB, Beta-blocker, MRA
Nabeta 2017 [16]	Japan	DCM	N/A	68	54 (12)	50/68	ACEI/ARB, Beta-blocker, MRA
Chimura 2017 [15]	Japan	DCM	WHO criteria/International Society and Federation of Cardiology	128	N/A	N/A	ACEI/ARB, Beta-blocker, MRA, diuretics, digoxin, amiodarone
Kubanek 2013 [7]	Czech Republic	RODCM	N/A	44	43 (11)	31/44	Angiotensin receptor neprilysin inhibitor, ACEI/ARB, Beta-blocker, MRA, diuretics, Sodium-glucose co-transporter inhibitors
Cho 2010 [14]	South Korea	NICM	Echocardiograph LVEF < 35%	79	LGE− n = 37: 55.27 (14.82) LGE+ n = 42: 57.40 (12.27)	48/79	Beta-blocker, ACEI/ARB, MRA, Oral inotropic agents
Merlo 2018 [20]	Italy	NICM	N/A	80	49 (39–58)	61/80	ACEI/ARB, Beta-blocker, MRA, diuretics, digoxin, warfarin

ACEI: angiotensin converting enzyme inhibitor; ARB: angiotensin receptor blocker; DCM: dilated cardiomyopathy; IQR: interquartile range; LGE: late gadolinium enhancement; LVRR: left ventricular reverse remodeling; MRA: mineralocorticoid receptor antagonist; NICM: non-ischemic cardiomyopathy; NIDCM: non-ischemic dilated cardiomyopathy; RODCM: recent onset dilated cardiomyopathy; WHO: World Health Organization. The values are expressed as the mean (standard deviation) or median (interquartile range). N/A: not applicable.

**Table 2:** Characteristics of included studies (information of LGE and LVRR).

Author Year	Baseline LVEF	LVRR Definition	LGE Measurement	LVRR %	LGE Absence		
					OR	95% CI (Lower Limit)	95% CI (Upper Limit)
Kan 2023 [25]	LVRR− (n = 53); 13.67 [11.36–19.84] LVRR+ (n = 41); 16.05 [11.27–21.71]	ΔLVEF ≥ 10% to a final value of >35% and ΔLVEDD ≥ 10%	5SD	43.6	2.99	1.21	7.41
Kinoshita 2022 [24]	37 (10)	ΔLVEF ≥ 10% and ΔLVEDV ≥ 10%	5SD	53.1	7.78	3.32	18.21
Xu 2021 [23]	25 (11)	ΔLVEF > 10% to a final value of ≥35% and ΔLVEDV > 10%	5SD	30.6	2.94	1.39	6.25
Kimura 2021 [22]	32.2 (9.5)	ΔLVEF ≥ 10% to a final value of >35% and ΔLVEDD ≥ 10%	N/A	N/A	5.56	1.39	20.00
Saito 2020 [21]	47.7 (15.1)	ΔLVEF ≥ 10% to a final value of >35% and ΔLVEDD ≥ 10%	N/A	45.5	3.23	1.01	11.11
Inui 2018 [19]	20.1 (10.2)	ΔLVEF ≥ 10%	Semiquantitative	66.7	1.21	0.27	5.40
Ehara 2018 [18]	30.2 (7.5)	ΔLVEF ≥ 10% and ΔLVEDD ≥ 10%	N/A	66.7	7.50	1.69	33.27
Barison 2018 [17]	35 (27–41)	ΔLVEDVi ≥ 10% and ΔLVEF ≥ 10%	6SD	31.0	2.98	1.05	8.42
Nabeta 2017 [16]	32 (8)	ΔLVEF ≥ 10% to a final value of ≥35% and ΔLVEDDi ≥ 10%	5SD	44.1	4.84	1.72	13.62
Chimura 2017 [15]	N/A	ΔLVEF ≥ 10% and ΔLVEDV ≥ 10%	N/A	59.4	2.30	1.02	5.17
Kubanek 2013 [7]	23 (7)	ΔLVEF ≥ 10% to a final value of >35% and ΔLVEDD ≥ 10%	2SD	45.5	2.00	0.55	7.24
Cho 2010 [14]	LGE− (n = 34); 28.26 (7.22) LGE+ (n = 42); 25.07 (9.14)	LVEF> 45%	N/A	32.9	8.79	2.43	31.88
Merlo 2018 [20]	33 (25–41)	ΔLVEF ≥ 10% or LVEF ≥ 50% and ΔLVEDD ≥ 10% or LVEDD ≤ 33 mm/m^2^	N/A	53.8	5.19	1.96	13.74

OR: odds ratio; CI: confidence interval; IQR: interquartile range; LGE: late gadolinium enhancement; LVEF: left ventricular ejection fraction; LVEDD: left ventricular end-diastolic dimension; LVEDV: left ventricular end-diastolic volume; LVRR: left ventricular reverse remodeling; SD: standard deviation. The values are expressed as the mean (standard deviation) or median (interquartile range). N/A: not applicable.

## Data Availability

The datasets used and/or analyzed during the current study are available from the corresponding author on reasonable request.

## Supplementary Materials

The following supporting information can be downloaded at: https://www.mdpi.com/article/10.3390/jcm14030895/s1, File S1: Search formula; Table S1: Newcastle-Ottawa Quality Assessment Scale Case Control Studies; Figure S1: Funnel plot of odds ratio of absence of LGE for the prediction of LVRR.
