# Supplementary material for: Late Gadolinium Enhancement Magnetic Resonance Imaging (MRI) for Predicting Left Ventricular Reverse Remodeling in Non-Ischemic Cardiomyopathy: A Systematic Review and Meta-Analysis
Source: J Clin Med. 2025 Jan 29;14(3):895. doi: 10.3390/jcm14030895 (PMC11818329; doi:10.3390/jcm14030895)
Supplement: Supplementary file 1 [file jcm-14-00895-s001.zip › jcm-3366547-supplementary.pdf]

Supplemental Material File S1

((((MRI OR CMR) OR "Magnetic Resonance Imaging") OR "Cardiac Magnetic Resonance")

AND

((LVRR OR "Left Ventricular Reverse Remodeling"))

AND

((DCM OR "Dilated Cardiomyopathy")))

Number of articles

-Pubmed 21

-WOS 29

-Cochrane library 1

Supplemental material Table S1. Newcastle - Ottawa Quality Assessment Scale Case Control Studies

**Study**	**Selection (Max=4)**	**Comparability (Max=2)**	**Exposure (Max=3)**	**Total Score**
Kan 2023	3	2	3	8
Kinoshita 2022	4	2	3	9
Xu 2021	4	2	3	9
Kimura 2021	4	2	3	9
Saito 2020	3	2	3	8
Inui 2018	3	2	3	8
Ehara 2018	3	2	3	8
Barison 2018	3	2	3	8
Nabeta 2017	3	2	3	8
Chimura 2017	4	2	3	9
Kubanek 2013	3	2	3	8
Cho 2010	2	2	3	7
Malro 2018	2	2	3	7

Supplemental material Figure S1. Funnel plot of odds ratio of absence of LGE for the prediction of LVRR.

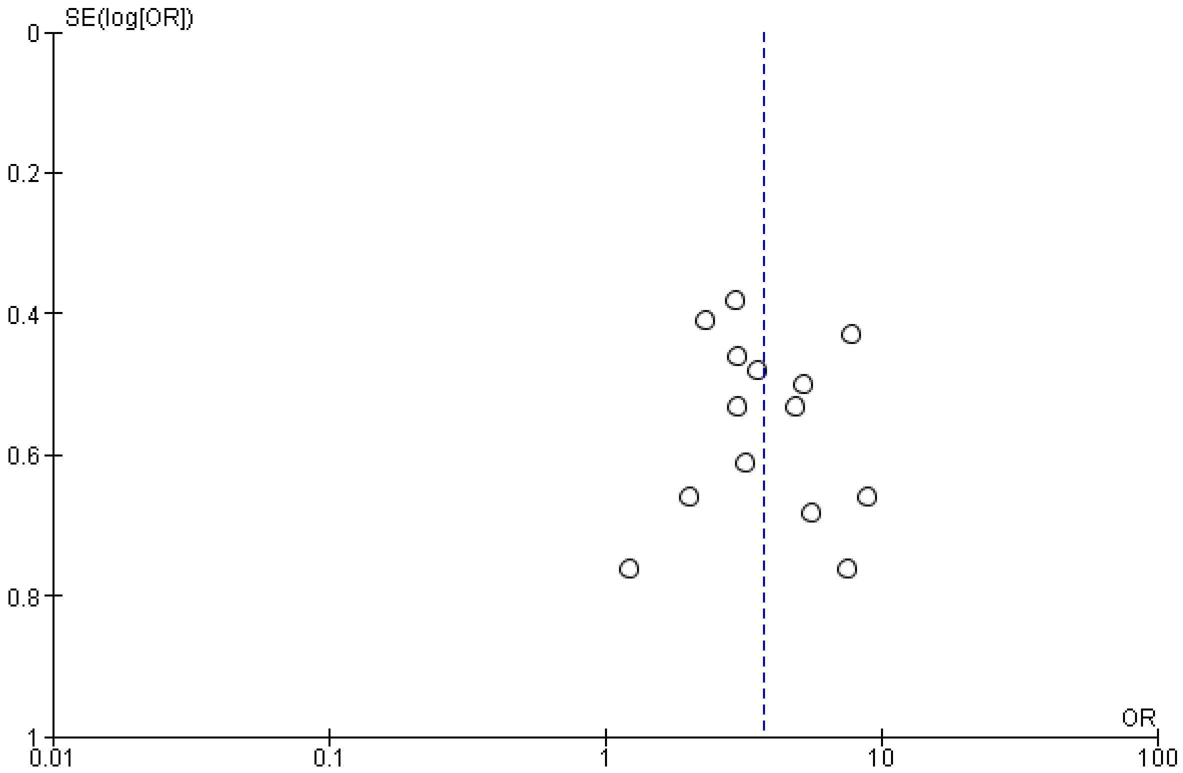

Begg's test yielded the following results.

Rank Correlation Test for Funnel Plot Asymmetry

Kendall's tau = 0.1341, p = 0.5093
